# Personal needs versus national needs: public attitudes regarding health care priorities at the personal and national levels

**DOI:** 10.1186/s13584-015-0010-2

**Published:** 2015-05-15

**Authors:** Giora Kaplan, Orna Baron-Epel

**Affiliations:** The Gertner Institute for Epidemiology and Health Policy Research, Sheba Medical Center, Tel Hashomer, 52621 Israel; School of Public Health, Faculty of Social Welfare and Health Studies, University of Haifa, Haifa, Israel

**Keywords:** Health policy, Health priorities, National health insurance, Health insurance, National survey, Public opinion

## Abstract

**Background:**

Many stakeholders have little or no confidence in the ability of the public to express their opinions on health policy issues. The claim often arises that lay people prioritize according to their own personal experiences and may lack the broad perspective necessary to understand the needs of the population at large. In order to test this claim empirically, this study compares the public’s priorities regarding personal insurance to their priorities regarding allocation of national health resources. Thus, the study should shed light on the extent to which the public’s priorities at the national level are a reflection of their priorities at the personal level.

**Methods:**

A telephone survey was conducted with a representative sample of the Israeli adult population aged 18 and over (n = 1,225). The public’s priorities were assessed by asking interviewees to assume that they were the Minister of Health and from this point of view allocate an additional budget among various health areas. Their priorities at the personal level were assessed by asking interviewees to choose preferred items for inclusion in their personal supplementary health insurance.

**Results:**

Over half of the respondents (54%) expressed different personal and national priorities. In multivariable logistic analysis, “population group” was the only variable found to be statistically significant; Jews were 1.8 times more likely than Arabs to give a similar response to both questions. Income level was of borderline significance.

**Conclusions:**

At least half of the population was able to differentiate between their personal needs and national policy needs. We do not advocate a decision-making process based on polls or referendums. However, we believe that people should be allowed to express their priorities regarding national policy issues, and that decision-makers should consider these as one of the factors used to determine policy decisions.

## Background

Responsiveness of public institutions to citizens’ needs and preferences is a central component of democratic theory and practice. In the health arena, similar to other sectors, there is a tendency towards listening to public opinion, especially regarding rationing and prioritizing health services [1-5]. Health technology assessment and prioritization are not just ‘technical’ processes restricted to professionals, because at some point during the process it becomes a choice between social values.

The need for rationing and prioritization is particularly evident in the Israeli health system since the inception of the National Health Insurance Law in 1995, whereby every citizen is entitled to a basic ‘basket of services’ distributed via membership in one of four health funds. This law reflects the value that there is a universal right to receive health care, however the decision about which services are included is subject to deliberation by a national multidisciplinary committee within the limits of a government-mandated budget. Their distribution, under circumstances of limited resources, has ethical consequences and requires societal approval in order to allocate medical treatment. Principles of distributive justice require consensus regarding the relevant issues that must be taken into consideration when allowing certain treatments for some, while withholding other treatments for others [6].

Some claim that the public should play a role in prioritization of health services [7], while others view public consultation as a tactical process aimed at guaranteeing that priorities and rationing are accepted [8]. However there are those who oppose involvement of the public altogether [7,9]. Certain philosophers express a strong traditional opposition to public policy based on social values, due to the fact that these values can be inherently unjust and a health system should not be governed according to the ‘tyranny of the masses’ [10]. When public values are opposed to standard ethical principles, for example when the public support discrimination against minorities, it is necessary to limit their influence on health policy [10].

Many professional stakeholders have no confidence in the ability of the public to express opinions on health policy issues. Among the arguments supporting this attitude, the claim often arises that lay people will choose priorities in health only according to their own personal experience or that of close family and acquaintances. They lack awareness of needs with which they are not familiar, and they lack a broader perspective in order to see the needs of a population at large.

Political scientists believe that issues about which people feel strongly will motivate political action to a great extent [11,12]. Among the major determinants of Americans’ opinions about policy matters, the self-interest perspective, which enjoys widespread currency in economics, political science, and psychology, suggests that Americans will support those policies that will help them maximize their short-term individual goals or interests [13]. However, surveys that seek to identify self-interest effects on policy preferences must frequently make simplistic assumptions about which characteristics identify those respondents whose self-interest would be served by particular policies [14].

According to these theories, the public’s priorities in health services should be guided by how they perceive the issues in relation to their personal needs (actual or anticipated), and a strong alignment between preferences at the personal and at the national level will be expected.

However, there are political researchers who have opposed this emphasis on self-interest. Their central claim is that the political influence of national circumstances is not a direct result of personal experience. While self-interest is often a weak predictor of policy preferences, much research shows that people rely heavily on partisan and ideological cues as shortcuts to form their opinions on complex policy issues [14].

Additional support for this self-interest hypothesis can be grounded in cognitive psychology, to which researchers in political science often refer [15,16]. People strive towards consistency between their various attitudes. When an inconsistent situation occurs, a cognitive rearrangement is made to reduce the inconsistency. Some models such as balance models, congruity approaches and dissonance theory have been developed to explain this situation [15,17]. Respondents expressing different priorities for national health care policies than for their individual health insurance would be in cognitive dissonance, unless they made a conscious, clear distinction between the levels, in which case the dissonance is resolved.

It is possible to extrapolate from these approaches to the field of health policy, asking if the areas to which the public grant priority regarding the national health budget is similar to their preferences for their personal health insurance.

Any successful attempt to change the health care system through public policy will need to account for public preferences. But what shapes public opinion toward health policy? Are policy preferences informed for the most part by citizens’ self-interest? Or do moral considerations about equality and fairness that lie at the heart of universal coverage also play an important role in public opinion? [14].

The preferences of the Israeli public regarding allocation of surplus resources in the national health care system, as well as their preferences for services to be included in their personal supplementary health insurance (which can be voluntarily purchased to supplement the “basket of services”) have been presented in a previous paper [18]. The aim of the current analysis is to assess to what extent the public’s priorities at the national level are a reflection of their priorities for their personal insurance, or to what extent do they differentiate between the national and personal levels.

## Methods

A telephone survey was conducted with a representative sample of the Israeli adult population aged 18 and over (N = 1,225). The sample design as well as the collection of data was conducted by The Cohen Institute for Public Opinion Research of Tel Aviv University. Interviews were conducted during June-August, 2008 and were carried out in Hebrew, Arabic and Russian. The response rate was 35.7% of the households in which someone answered the telephone. The distribution of the sample very closely represents that of the Israeli population aged 18 and over (Central Bureau of Statistics) with regard to age, gender, education, household income, self-evaluation of health status, and Health Maintenance Organization (HMO) membership. However, compared to the Israeli population the sample over-represented Arabs (25.2% vs. 16.1%) and those with supplementary health insurance (75% vs. 67%).

Two versions of the questionnaire were used to overcome the limitation of the length of the questionnaire for a telephone survey. In order to achieve a representative sample of the Israeli population, for each version approximately 600 participants was randomly assigned. This approach was based on the principles of Partial Questionnaire Design method (PQD) [19,20].

### Variables

The public’s priorities at the national level were assessed by asking interviewees to imagine that they were the Minister of Health and as such to rate on a four-level scale the extent to which they would allocate a surplus budget to each of the items presented. The items on one version of the questionnaire included: screening tests for early disease detection, cardiac rehabilitation, subsidized supplemental insurance for the poor, mental health care, fertility treatments, and alternative medicine. The second version of the questionnaire included: nursing care for the frail elderly, programs for preventive medicine and health promotion, dental care, additional staff for primary clinics, and building a new hospital. An additional question asked interviewees to indicate their top priority.

The public’s priorities at the personal level were assessed by asking interviewees to rate to what extent they would include each of the items noted in their personal supplemental health insurance. From the national level lists certain items were deleted (“fertility treatments” and “subsidized supplemental insurance” were deleted from the first version; “additional clinic staff” and “building a new hospital” from the second version). These were replaced by “getting a second opinion” in the first version, and “cosmetic surgery” and “hospitalization in a private hospital” in the second version. Interviewees were again asked to rate each item on a four-level scale and finally to choose their highest priority.

Independent variables included: age, gender, population groups (Jews, Arabs), education, household income, employment status (salaried, self-employed, pensioner, unemployed, homemaker) and family status (single-no children, single-parent family, married with children, married without children). Health-related variables included: self-evaluation of health status, degree of exposure to the health system (hospitalized during the last year and/or 5+ visits to a primary physician, 1-4 visits, and no contact with the medical system during last year), HMO (under Israeli National Health Insurance every citizen is enrolled in one of four HMOs), having supplementary health insurance and having additional private health insurance policies.

### Statistical analysis

For each item on the list of health services, the percentage of respondents’ agreement on both personal and national levels was calculated in two manners: (1) rating of each item and (2) the first priority. Chi-square test was used for univariate analysis. In order to identify personal characteristics of respondents who did or did not differentiate between the national and the personal level, a multivariable logistic regression analysis was performed including most independent variables.

Data analyses were performed using the 9.13 release of SAS PC computer software.

## Results

As previously published, the public’s top priorities were “screening tests for early disease detection” and “nursing care for the frail elderly” in both the distribution of national resources and in personal insurance [18].

Most areas having a high degree of public support received similar ratings at the national and personal levels (Table 1). “Mental health care” was the category with the highest proportion of the public choosing it only at the national level while not for personal insurance.

**Table 1:** The percentage that chose to award to each service the extra budget and to include it in the personal insurance*

	**Chose at both levels**	**Chose only in personal insurance**	**Chose only for national budget**	**Not choose**
Screening tests for early detection of disease	86.5	4.6	6.2	2.7
Nursing care	81.6	3.0	11.3	4.1
Cardiac rehabilitation	83.5	5.5	7.4	3.6
Preventative medicine and health promotion	64.4	6.0	14.6	15.0
Dental care	54.0	14.3	14.5	17.2
Mental health care	53.8	5.1	29.3	11.8
Alternative medicine	41.1	13.5	13.5	31.9

*“chose” includes 2 categories: ’I would certainly choose’ and ’It is likely that I would choose’. “Did not choose” includes two statements: ’I would certainly not choose’ and ’It is likely that I would not choose’. Included in the table are only the participants that answered both questions. The areas included are those that appeared in both questions.

Tables 2 and 3 show respondents’ first choice at the national level by their first choice on their personal insurance for each version of the question. Over half of those who chose as first priority for their personal insurance “screening tests for early detection of disease ”, “mental health care” (Table 2), “nursing care” and “health promotion” (Table 3), chose to award the surplus budget at the national level to the same area. This was also found in the case of the item regarding hospitals, for which the items were different on the personal (“hospitalization in a private hospital”) and national (“building a new hospital”) levels. In contrast, almost 70% of those who chose “cardiac rehabilitation” for their personal insurance, awarded surplus national budget to a different item, and almost all of those who chose “alternative medicine” or “dental care” for their personal insurance, did not give preference to these items at the national level. People who chose “second opinion” for their own insurance, tended to give surplus national budget to “screening for early disease detection” or to “subsidizing supplemental insurance for the poor” (Table 2).

**Table 2:** **Percentage choosing each area as first priority for surplus budget by first priority for personal insurance - Version 1 of questionnaire (N = 609)**

**First priority for surplus budget**	**First priority for personal insurance**					
	**Screening tests**	**Cardiac rehabilitation**	**Mental health care**	**Alternative medicine**	Second opinion*	**No response**
	**n (%)**	**n (%)**	**n (%)**	**n (%)**	**n (%)**	**n (%)**
Total	278 (100.0)	142 (100.0)	65 (100.0)	38 (100.0)	30 (100.0)	56 (100.0)
Screening tests	155 (55.8)	29 (20.4)	9 (13.8)	9 (23.7)	7 (26.7)	10 (17.9)
Cardiac rehabilitation	30 (10.8)	45 (31.7)	5 (7.7)	5 (13.2)	4 (13.3)	5 (8.9)
Mental health care	14 (5.0)	14 (9.9)	35 (53.9)	5 (13.1)	2 (6.7)	8 (14.3)
Alternative medicine	0	3 (2.1)	2 (3.1)	3 (7.9)	0	0
Subsidized supplemental insurance for the poor*	44 (15.8)	16 (11.3)	6 (9.2)	10 (26.3)	[6 (20.0)]	8 (14.3)
Fertility treatments*	20 (7.2)	23 (16.2)	5 (7.7)	4 (10.5)	[5 (16.7)]	3 (5.4)
No response	15 (5.4)	12 (8.4)	3 (4.6)	2 (5.3)	5 (16.7)	22 (39.3)
Total:						
Same item +	155	45	35	3	--	
Different item	123	97	30	35	18**	

*These items were not response options to both questions (personal insurance and supplementary budget).

**11 responses marked [ ] were not included because the response combination related to two items not included in both questions.

*+ Percentage choosing the same item = 44.0% (238/541).*

**Table 3:** **Percentage choosing each area as first priority for surplus budget by first priority for personal insurance - Version 2 of questionnaire (N = 616)**

***First priority for surplus budget***	***First priority for personal insurance***					
	**Nursing care**	**Preventive medicine and health promotion**	**Dental care**	Cosmetic surgery*	Hospitalization in private hospital*	**No response**
	**n (%)**	**n (%)**	**n (%)**	**n (%)**	**n (%)**	**n (%)**
Total	272 (100.0)	121 (100.0)	102 (100.0)	5	74 (100.0)	42 (100.0)
Nursing care	177 (65.1)	20 (16.5)	27 (26.5)	3	15 (20.3)	10 (23.8)
Preventive medicine and health promotion	27 (9.9)	61 (50.4)	16 (15.7)	1	7 (9.5)	6 (14.3)
Dental care	3 (1.1)	2 (1. 6)	16 (15.7)		4 (5.4)	2 (4.8)
Additional staff for primary clinics*	12 (4.4)	8 (6.6)	8 (7.8)		4 (5.4)	1 (2.4)
Building a new hospital*	45 (16.5)	25 (20.7)	32 (31.4)	[1]	[43] (58.1)	7 (16.7)
No response	8 (2.9)	5 (4.1)	3 (2.9)		[1] (1.3)	16 (38.1)
*Total:*						
Same item +	177	61	16			
Different item	95	60	86	4	27**	

*These items were not response options to both questions (personal insurance and supplementary budget).

**48 responses marked [ ] were not included because the response combination related to two items not included in both questions.

*+ Percentage choosing the same item = 48.3% (254/526).*

Combining the findings of the two versions (Table 2 and 3), a total of 492 participants (46%) chose the same item for first priority at both levels – personal insurance and national budget.

Population group was the only independent variable significantly related to choosing the same item at both levels. Multivariable logistic analysis was conducted including all variables with a level of significance under 0.2 (Table 4), and only this variable was statistically significant, with Jews 1.8 times more likely than Arabs to give the same response to both questions. Borderline significance was found for income, with those of average income having a stronger tendency to prioritize different services at the personal and national levels than those with highest income.

**Table 4:** **Characteristics of respondents who chose the same item as first priority for their personal insurance and for national budget: Logistic models (reference group: not the same choice for both questions)**

**Version 1 of questionnaire (n = 453)**			
	**Odds ratio**	**95% confidence limits**	**p-value**
*Age group:*			
18-29	1.57	0.69 – 3.57	0.681
30-49	1.81	0.83 – 3.96	0.195
50-64	1.56	0.73 – 3.33	0.685
65+	1.00		
*Gender:*			
Men	1.00		
Female	0.87	059 – 1.29	0.497
*Income:*			
Below average	1.00		
Average	0.97	0.59 – 1.59	0.726
Above average	1.10	0.67 – 1.79	0.619
*Employment status:*			
Salaried			
Self-employed			
Unemployed			
*Health status:*			
Excellent	1.17	0.54 – 2.54	0.773
Very good	0.94	0.43 – 2.02	0.346
Good	1.40	0.68 – 2.86	0.191
Not so good, Bad	1.00		
*Exposure to the health system:*			
High	0.86	0.51 – 1.44	0.712
Low	1.00		
No direct exposure	0.91	0.52 – 1.58	0.947
**Version 2 of questionnaire (n = 497)**			
*Age group:*			
18-29	0.78	0.33 – 1.88	0.586
30-49	0.89	0.39 – 2.06	0.789
50-64	0.93	0.42 – 2.50	0.849
*Income:*			
Below average	1.18	0.74 – 1.86	0.485
Average	1.61	0.98 – 2.67	0.061
Above average	1.00		
*Employment status:*			
Salaried	0.77	0.44 – 1.35	0.356
Self-employed	1.00		
Unemployed	0.71	0.24 – 2.09	0.536
*Population group:*			
Arabs	1.00		
Jews	1.81	1.14 – 2.88	**0.011**

## Discussion

In this study participants were asked to choose priorities separately for their personal needs (i.e. supplementary health insurance) and at the national level (i.e. allocating surplus national budget). Doubts have been raised in the literature about establishing healthcare priorities based on population surveys, the main reservation being the concern that lay people’s opinions are shaped primarily by their personal needs and experiences. Based on this “self-interest” assumption, the individual will always prefer to promote issues that will be of personal benefit [21]. This approach is supported by psychological dissonance theory and conventional political science theory [11]. This is supported by the finding that almost half of those interviewed in this study made a similar choice at both levels.

However, over half of the interviewees made different choices at the personal and national level. Analysis of preferences at the national level according to the choice made for the personal insurance indicates that the majority of those who chose “cardiac rehabilitation”, “dental health care” and “alternative medicine” as their first priority for personal insurance selected a different area for surplus national budget. This was also the case for almost half of those who chose “screening tests”, “mental health care”, “nursing care for the elderly” and “health promotion” as the first personal priority.

Explanations for these findings may be found in the claim of some political scientists that people are able to develop political preferences without any direct personal interest, or even in contradiction to their personal interest [22]. The political response of citizens is mediated by their judgment of the collective situation. For example, a rise in the unemployment rate, or in inflation, or the deterioration of public services, is judged independently from the individual’s personal situation [11]. People’s judgment of the situation includes an ideological dimension that influences preferences and opinions regarding issues that don’t touch them directly or personally. For example, men may support or be against abortion, even if they believe that their wives and daughters will never need to undergo one. At times the number of people who will be significantly influenced by a particular policy is much smaller than the number of those who support this policy. In other words, a person has the ability to create preferences even in the absence of immediate personal interest [22].

In this empirical study we found that over half of the population differentiates in health policy issues between social or national policy and their personal needs. When asked about national policy, they seem to be influenced by additional considerations beyond their personal experience (e.g. social solidarity). Furthermore, it may be that some of the subjects who chose the same priority at both levels actually believe that the national interest is the same as their own personal interest, thus for them there is a coincidence of private and public interests. Although we were unable to test this hypothesis, considering this aspect, it is likely that the 54% of the public who differentiated between personal and national priorities is a minimal estimate, and their proportion in the population may be even higher.

The only socio-demographic variable that characterized those with a stronger tendency to differentiate between personal and national levels was population group. Arab respondents were more likely to make different priorities at the two levels. A possible explanation for this is that the Arab population, being a minority with a different cultural background than that of the majority Jewish population, perceives their needs as different from the needs of the majority. Therefore at the national level they tend to perceive the majority’s needs independently from their own. A more in-depth exploration of this hypothesis is recommended.

## Conclusion

We do not advocate a decision-making process based on polls or referendums. However we believe that citizens are able to express their opinion on national policy issues as distinct from their personal needs. This information is important to decision-makers as one of the factors that should be considered when making policy decisions.
